# Comparative analysis of hand-held and stationary ultrasound for detection of sarcopenia in acutely hospitalised older adults—a validity and reliability study

**DOI:** 10.1007/s41999-024-01021-x

**Published:** 2024-07-20

**Authors:** Jeppe Grabov Phillip, Lisbeth Rosenbek Minet, Siri Aas Smedemark, Jesper Ryg, Karen Andersen-Ranberg, Kristoffer Kittelmann Brockhattingen

**Affiliations:** 1 Department of Geriatric Medicine, Odense University Hospital, Svendborg, Denmark; 2 Department of Geriatric Medicine, Odense University Hospital, Odense, Denmark; 3 Geriatric Research Unit, Department of Clinical Research, University of Southern Denmark, Odense, Denmark; 4 Department of Clinical Research, University of Southern Denmark, Odense, Denmark

**Keywords:** Geriatric, Muscle assessment, Ultrasonography, Hand-held ultrasound, Older adults, Sarcopenia

## Abstract

**Background and purpose:**

Sarcopenia is a growing health concern among geriatric patients. Early diagnostics is importance to intervene and better muscle status and thus physical function. Ultrasound can be a valuable tool for patient-near diagnostics of sarcopenia. In recent time, ultrasound devices have evolved from larger stationary devices to minor hand-held devices that are more portable. However, the literature lacks research comparing quality of the different devices. The purpose of this study was to compare hand-held ultrasound (HH-US) to stationary ultrasound (S-US) in muscle assessment for detection of sarcopenia in acutely hospitalized older adults.

**Methods:**

A cross-sectional study using a convenience sample of acutely admitted older patients examined with both HH-US and S-US within a single session by the same examiner. Image analysis was performed using ImageJ, and was conducted by two raters: Rater 2 an experienced US examiner and Rater 1 an US examiner who received training from Rater 2. The Ultrasound sarcopenia index (USI) was used for evaluating sarcopenia. Validity and reliability of HH-US were analyzed using Cohen’s Kappa and Student’s *t*-test.

**Results:**

21 participants (mean age 83.4 years, 52% female). Results showed “substantial” intra-rater reliability (*κ* = 0.77 for Rater 1) and ‘near-perfect’ validity (*κ* = 0.92 for Rater 2). Inter-rater comparisons revealed no significant differences (*p* < 0.05).

**Conclusion:**

HH-US is a potential method for detection of sarcopenia in acutely hospitalized older adults.

## Key summary points

**Aim:**

To compare hand-held ultrasound (HH-US) to stationary ultrasound (S-US) in muscle assessment for detection of sarcopenia in acutely hospitalised older adults.

**Findings:**

Rater 1 had ‘substantial’ agreement between HH-US and S-US (*κ* = 0.77), whereas Rater 2 had ‘almost perfect’ agreement (*κ* = 0.92). Finally, no significant differences were seen on any US variables among the two raters when comparing the results from both HH-US and S-US.

**Message:**

HH-US scanners could be feasible, valid, and reliable for detection of loss of muscle mass associated with sarcopenia in acutely admitted older patients, in the hands of an experienced examiner.

## Introduction

Sarcopenia is an escalating health concern with implications for mobility, independence, risk of falls, heightened healthcare costs, increased risk of hospitalization, and increased mortality [1]. The aging process induces structural changes in skeletal muscle, predisposing individuals to sarcopenia, as do other prevalent factors among older adults, such as diseases, physical inactivity, and malnutrition [1].

Early disease detection is paramount for timely intervention and healthcare resource allocation. Current methods for diagnosing sarcopenia, such as dual-energy X-ray absorptiometry (DXA), bioimpedance analysis (BIA), CT, and MRI, are hindered by high costs, being stationary, and the need for specialized personnel [2], besides needing transportation of the patient. B-mode Ultrasonography (US) emerges as a promising tool for assessing skeletal muscle quantity and quality [3, 4]. This technique is valid, reliable, cost-effective, and easily accessible, particularly in geriatric settings [5, 6]. To date, the majority of clinical ultrasound scanners are stationary (S-US), limiting their application in dynamic healthcare settings. The advent of hand-held ultrasound scanners (HH-US), bypass these limitations, particularly in settings with older immobile populations, due to good portability, affordability, and ease of use. While previous studies have explored the feasibility of HH-US in clinical practice [8, 9], studies are lacking on the validity and reliability in exploring sarcopenia in high-risk populations such as acutely hospitalized older adults. Furthermore, the recent published ultrasound sarcopenia index for muscle wasting (USI) enables stratification of individuals according to muscle status into the following conditions: non-sarcopenic, pre-sarcopenic, moderately sarcopenic, sarcopenic, and severely sarcopenic [7]. Therefore, the aim of this study is to describe the validity and reliability of HH-US for muscle assessment by the USI in acutely hospitalized older adults, when compared to S-US.

## Materials and methods

### Study design and settings

We made a single-center cross-sectional study at the Department of Geriatric Medicine, Odense University Hospital Svendborg, Denmark. The department holds 32 beds with acutely hospitalized older patients for various illnesses, e.g., falls, infections, and confusion. Participants were recruited using convenience sampling from November 28 to December 16, 2022 and were eligible for inclusion if they met the following criteria:

1. Aged 65 years or older
2. Able to communicate with the researcher
3. Not in a delirious condition as measured by cognitive assessment method (CAM), and/or undiagnosed with dementia.

Exclusion criteria:

1. Younger than 65 years of age.
2. In delirious state
3. Diagnosed with dementia
4. Bilateral femur amputation of the extremities

Two different US scanners were used; (A) HH-US (Vscan Air, GE Healthcare, USA) equipped with a 3–12 MHz linear probe combined with an iPhone 13 (Apple, California, United States) and (B) S-US (GE Venue R2, GE Healthcare) equipped with a 8–13 MHz linear array probe used as the gold standard.

All participants were scanned with both scanners in a random order on the same visit by the same examiner (Rater 1: J.G.P.). Prior to data collection, the examiner (J.G.P.) had received intensive training and supervision from a very experienced medical doctor (Rater 2: K.K.B.) with > 7 years of experience, in acquiring and analyzing US images.

### Ultrasound examination

**Fig. 1:**
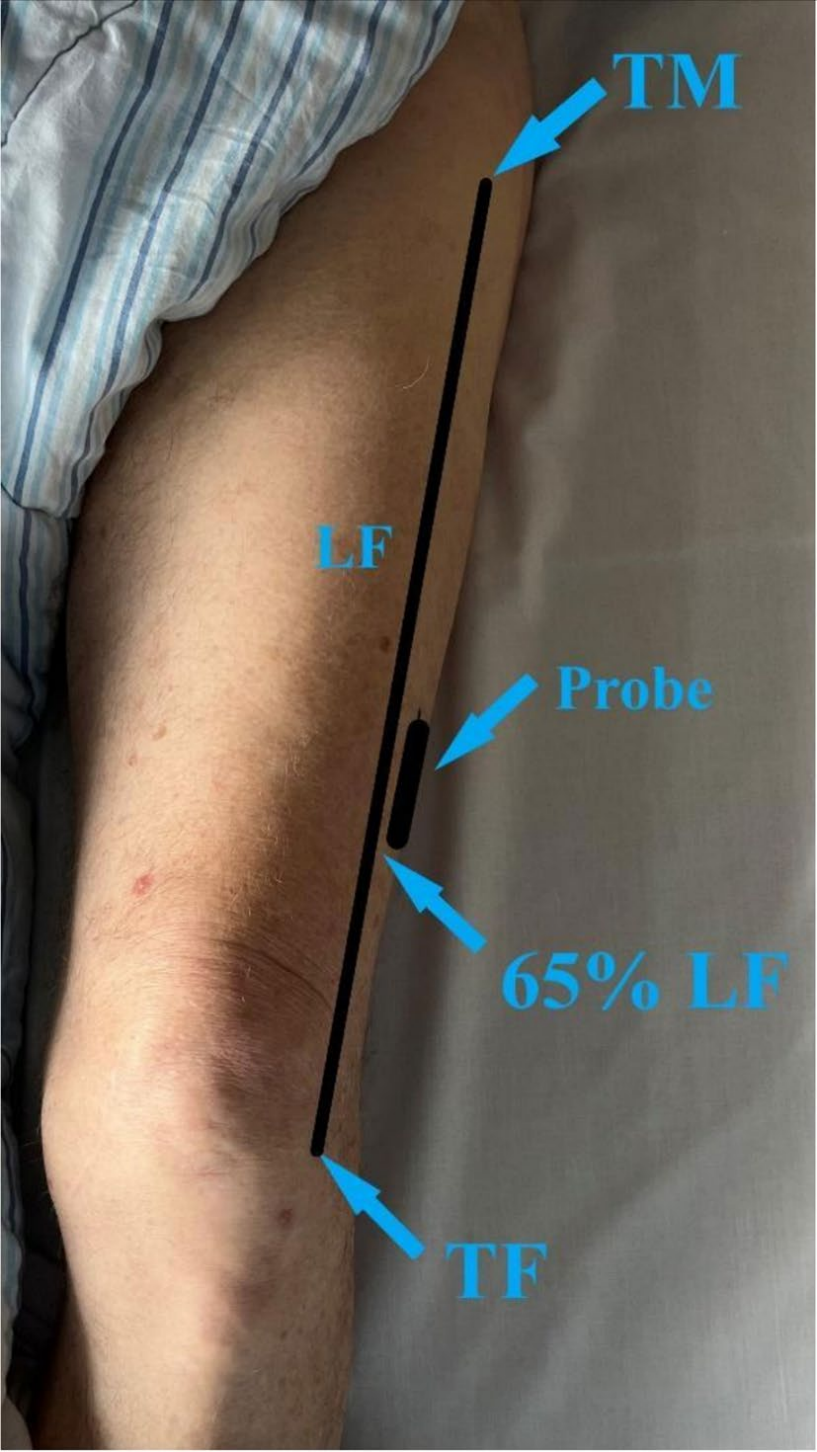
Anatomical image of the lower limb showing key reference points; FL; Total femur length.TM; Mid-proximal part of the greater trochanter. TF; Distal border of the tibio-femorale joint space

The US examination followed a protocol proposed by Narici et al. [7]. In short, participants, having rested for 5 min, lay supine with extended knees in resting position during the scan. Scans targeted the distal third of the muscle vastus lateralis (VL) on dominant leg (identified by asking the patient), marking 65% of the femur length (LF) from the greater trochanter (TM) to the distal border of the tibio-femorale joint space (TF) (7). This spot, aligned with the probe’s distal edge, guided the perpendicular placement of the transducer along the mid-sagittal axis of the VL (Fig. 1). Three longitudinal images were captured with both scanners repositioning after each image using sufficient contact gel and applying minimal pressure.

### Image analysis

**Fig. 2:**
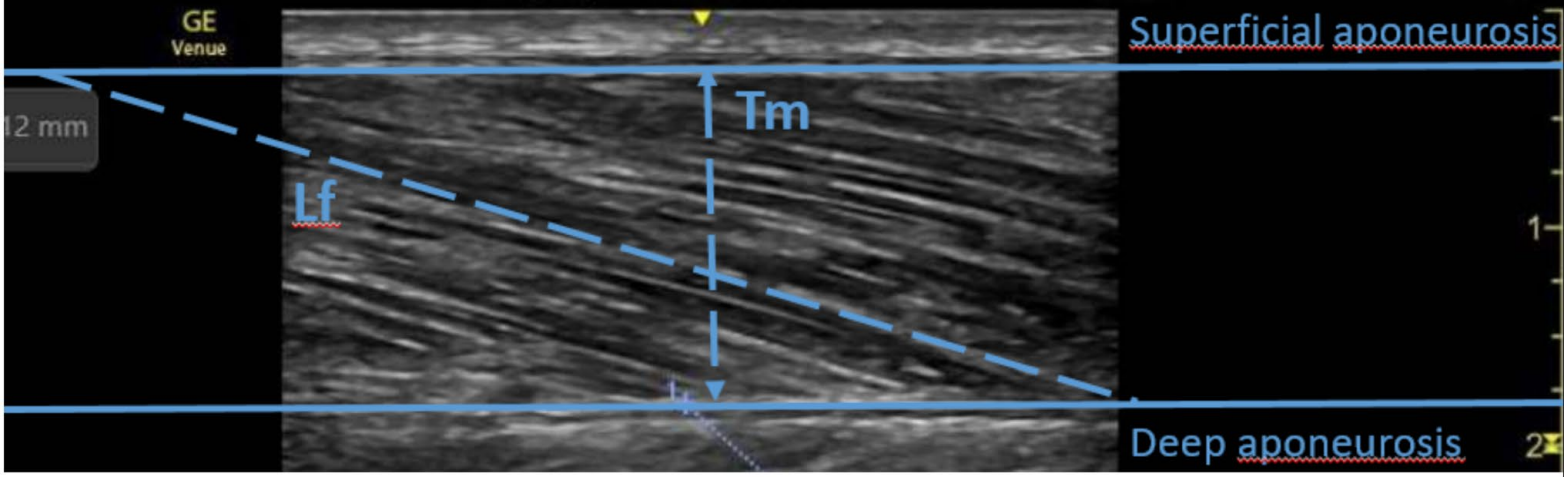
Longitudinal ultrasound image of the muscle vastus lateralis (VL), showing muscle thickness (Tm) and fascicle length (Lf)

"NIH ImageJ" software (version 1.42q) was used to calculate muscle thickness (Tm) and fascicle length (Lf) [7]. Tm was measured orthogonally between aponeuroses at mid-image, while Lf spanned between fascicle insertions on superficial and deep aponeuroses (Fig. 2). The mean of three measurements for each parameter was performed. The Lf/Tm ratio served as USI for evaluating sarcopenia [7]. Image analysis was performed independently by both raters at separate locations.

### Sample size

To our knowledge, no studies have published minimal detectable differences regarding measures of muscle architecture in the lower extremities of older adults. A previous study with an objective close to ours—albeit not the same—reported a sample size of 16 participants [10]. Since this study was the closest one to ours, we used this as a measure for choosing a sample size. Therefore, it has not been possible to estimate a sample size for this study. However, we aimed of a sample size of at least 20 participants for the current study.

### Statistical analysis

Demographic characteristics of the participants were summarized using the descriptive analysis. The Shapiro–Wilk test was used to evaluate the normality of data with results presented as means (± SD). The validity and intra-rater reliability of US measurements was evaluated using Cohen’s Kappa (*κ*), with values categorizing agreement from ‘none’ (0.01–0.20) to ‘almost perfect’ (0.81–1) [11]. Inter-rater reliability differences were tested via Student’s *t* test. Significance was set at *p* < 0.05, with data management in Microsoft Excel and analysis performed in R-Statistics.

## Results

In total, 21 participants (11 women) were recruited and had the following characteristics: A mean age of 83.4 (10.7) years, a mean BMI of 21 (4.0), a mean Barthel-100 of 65 (27), and height level of comorbidity with a mean CCI of 6 (1.6) (Table 1). In addition, pneumonia (including viral pneumonia) was the most common cause for admission (9 participants), followed by falls (3) and urine retention (2) (Table 1). In total, 11 participants had infection as primary cause of admission. Musculoskeletal causes, such as falls, and pain management and mobilization was found in 4 participants (characteristics and reason for admission shown in Table 1). Data regarding validity and intra-rater reliability, results presented as mean with SD, of US measurements of Tm, Lf, and USI of HH-US compared with S-US for both raters are presented in Table 2. Overall, no significant differences were seen regarding the different US variables nor USI between the two US scanners, except for Rater 2 Lf. Rater 1 had ‘substantial’ agreement between HH-US and S-US (*κ* = 0.77), whereas Rater 2 had ‘almost perfect’ agreement (*κ* = 0.92). Finally, no significant differences were seen on any US variables among the two raters when comparing the results from both HH-US and S-US.

**Table 1:** Participants characteristics

*n*: 21	
Age (years), mean (SD)	83.4 (10.7)
Women (*n*), (%)	11 (52.4%)
Height (cm), mean (SD)	170.0 (10.0)
Weight (kg), mean (SD)	71.0 (15.9)
BMI (kg/m^2^), mean (SD)	21 (4.0)
Barthel index 100 (Shahs version), mean (SD)	65.4 (27.3)
Charlson comorbidity index, mean (SD)	6.0 (1.6)
Primary reason for admission (%)	
Pneumonia (*n*)	7 (33.3)
Virus pneumonia (*n*)	2 (9.4)
Urinary retention (*n*)	2 (9.4)
Urinary tract infection (*n*)	1 (4.8)
Infection unknown origin (*n*)	1 (4.8)
Confusion (*n*)	1 (4.8)
Fall (*n*)	3 (14.3)
Hypertension (*n*)	1 (4.8)
Social admission (*n*)	1 (4.8)
Constipation (*n*)	1 (4.8)
Pain management and mobilisation, (*n*)	1 (4.8)

Data are presented as mean values ± standard deviation or *n* (%)

*BMI* body mass index

**Table 2:** Validity and reliability of HH-US compared to S-US

a Validity and intra-rater reliability of ultrasound measurements for HH-US and S-US for rater 1			
Variable	HH-US	S-US	*p*
VL Tm (cm), mean (SD)	1.74 (0.35)	1.72 (0.36)	0.62
VL Lf (cm), mean (SD)	6.94 (0.62)	6.98 (0.67)	0.82
			*κ*
USI, mean:	Pre-sarcopenic	Pre-sarcopenic	0.77

b Validity and intra-rater reliability of ultrasound measurements for HH-US and S-US for rater 2			
Variable	HH-US	S-US	*p*
VL Tm (cm), mean (SD)	1.78 (0.38)	1.75 (0.35)	0.52
VL Lf (cm), mean (SD)	7.31 (1.48)	7.72 (1.37)	0.003
			*κ*
USI, mean	Pre-sarcopenic	Pre-sarcopenic	0.92

c Inter-rater reliability of ultrasound measurements HH-US and S-US for rater 1 and rater 2			
Variable	Rater 1 Mean (SD)	Rater 2 Mean (SD)	*p*
VL Tm(cm) HH-US	1.74 (0.35)	1.78 (0.38)	0.91
VL Tm (cm) S-US	1.72 (0.36)	1.75 (0.35)	0.87
VL Lf (cm) HH-US	6.94 (0.62)	7.31 (1.48)	0.28
VL Lf (cm) S-US	6.98 (0.67)	7.72 (1.37)	0.06
USI HH-US	Pre-sarcopenic	Pre-sarcopenic	
USI S-US	Pre-sarcopenic	Pre-sarcopenic	

Data are presented as mean values ± standard deviation

*HH-US* hand-held ultrasound, *κ* Cohen’s Kappa coefficient, *Lf* fascicle length, *S-US* stationary ultrasound, *Tm* muscle thickness; *USI* ultrasound sarcopenia index, *VL* muscle vastus lateralis

## Discussion

We found an almost “perfect agreement” between HH-US and S-US, when image analysis is carried out by an experienced investigator. Furthermore, we found no significant differences between the two raters’ measurements of US variables and USI from HH-US compared to S-US. Hence, HH-US is sufficient for muscle assessment in the hands of an experienced examiner.

The reported results of US variables (Tm and Lf) are in line with a recent study in older adults [12]. However, our findings cannot be directly compared to prior studies as it is the first to describe HH-US versus S-US use in assessing sarcopenia among geriatric inpatients.

### Strengths and limitations

Our study has several strengths. First, the design ensured blinding of results between the two raters limiting potential bias and heightening result credibility [13]. Second, all scans were conducted by the same examiner (J.G.P) ensuring uniformly approach and no differences in quality was experienced. In addition, we made sure the US protocol used only required a minimum level of ultrasound training [14], thus limiting potential errors and strengthen the quality of repeated results. Third, generalizability of the results was strengthened by examining a highly prevalent hospital population, hence making the study relevant for clinical practice.

Our study also has several limitations. First, it was carried out as a single-center study, which may limit applicability to other clinical settings. Second, the scans were performed by a novice examiner who had received US training from an experienced US investigator, which may impair the imaging quality and thereby the interpretation of the USI graduation. Third, we used a small sample size. This may weaken the power of the study and therefor the results. Fourth, due to the general reduced transducer’s window width of the HH-US device, Lf extrapolation was generally necessary, which might result in measurement errors [15]. This could potentially lead to mis-graduation of participants according to the USI. However, when comparing the mean Lf values of HH-US with S-US, no significant differences were found, except for Rater 2’s Lf values. Despite this important limitation, it did not affect overall USI scores when comparing the two scanners.

## Conclusion

This study shows that HH-US scanners could be feasible, valid, and reliable for detection of loss of muscle mass associated with sarcopenia in acutely admitted older patients, when compared to S-US in the hands of an experienced examiner. Their portability, affordability, and ease of use could enhance ultrasound accessibility in hospital settings and may enhance clinical practices for sarcopenia detection in older adults. Further studies are necessary to validate HH-US efficacy across different muscles and broader populations.
